# Antioxidant Properties of Selenophene, Thiophene and Their Aminocarbonitrile Derivatives

**DOI:** 10.3390/antiox6020022

**Published:** 2017-03-24

**Authors:** Levon A. Tavadyan, Zaruhi H. Manukyan, Lusik H. Harutyunyan, Makich V. Musayelyan, Adrine D. Sahakyan, Hakob G. Tonikyan

**Affiliations:** Aram Bagrat Nalbandyan Institute of Chemical Physics, National Academy of Sciences, 5/2 Sevak Street, Yerevan 0014, Armenia; zaramanukyan@mail.ru (Z.H.M.); lusik@ichph.sci.am (L.H.H.); makich@ichph.sci.am (M.V.M.); adrine@ichph.sci.am (A.D.S.); htonikyan@yahoo.com (H.G.T.)

**Keywords:** selenophene, thiophene, aminocarbonitrile derivatives, antioxidant, antiperoxyradical capacity, CV, DPV and SWV voltammetry

## Abstract

The oxygen radical absorbance capacity (ORAC) method was used to detect the antiperoxyradical ability of organoselenium compounds: selenophene and its derivative, 2-amino-4,5,6,7-tetrahydro-1-selenophene-3-carbonitrile (ATSe); while as a comparison, the sulfur-containing analogue of selenophene—thiophene and its derivative—2-amino-4,5,6,7-tetrahydro-1-thiophene-3-carbonitrile (ATS)—was selected. Cyclic voltammetry (CV), differential pulse voltammetry (DPV) and squarewave voltammetry (SWV) methods were used to determine the redox characteristics of organoselenium and organosulfur compounds. The antiradical activity and capacity of the studied compounds were also measured by using stable radical 2,2ʹ-diphenyl-1-picrylhydrazyl (DPPH). Detected anodic peaks of the oxidation of selenophene, thiophene and their derivatives in CV, DPV and SWV in the interval of −1200 ÷ (+1600) mV potentials in regard to the Ag/Ag^+^ medium of acetonitrile prove the presence of antiperoxyradical activity in regard to oxidizers, i.e., peroxyradicals. The chemical mechanism of the antiperoxyradical ability of selenophene, thiophene and their organic derivatives is proposed.

## 1. Introduction

Studies of the chemical mechanism of organoselenium antioxidant action are urgent. In recent years, special interest has been given to selenium-containing compounds as antioxidants, regulating free radical reactions in the organism, and significantly affecting its state [1,2,3,4,5,6,7]. They neutralize, and “scavenge” free radicals and other reactive oxygen-containing species (ROS), the excess generation of which causes many pathological conditions known as oxidative stress [8,9,10,11,12]. Meanwhile, the role of organoselenium compounds in in vivo, and in vitro conditions is not fully studied.

**Scheme 1:**
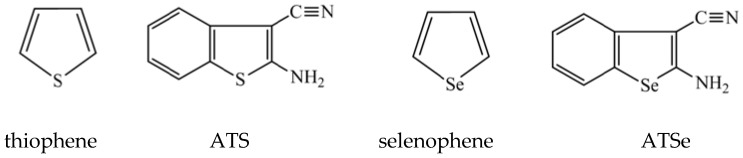
Structural formulas of the studied seleno- and sulfo-organic compounds. ATS: 2-amino-4,5,6,7-tetrahydro-1-thiophene-3-carbonitrile, ATSe: 2-amino-4,5,6,7-tetrahydro-1-selenophene-3-carbonitrile.

The selection of seleno- and sulfo-organic compounds, such as thiophene, selenophene and their derivatives: aminocarbonitriles (Scheme 1), was conditioned by the following reasons:

1. ATS (2-amino-4,5,6,7-tetrahydro-1-thiophene-3-carbonitrile) and ATSe (2-amino-4,5,6,7-tetrahydro-1-selenophene-3-carbonitrile) are the basic compounds for the synthesis of a number of bioactive heterocyclic compounds [1,2]. On the basis of these compounds, drugs with antiviral and antibacterial properties [1,2], as well as analogs of drugs to treat Alzheimer's disease were obtained [3].
2. In recent years, preparative available methods for the synthesis of considered compounds were revealed using ultrasound (US) and microwave radiation, especially for the selenophene derivatives [3].
3. It was also supposed that the presence of sulfo- and seleno-groups in the conjugated molecular system will reduce the unpleasant odor of such compounds, which is an urgent problem. It can be assumed, for example, by comparing Ebselen (entered into medical practice) with diethyl selenide [13,14]. Simultaneously, the presence of aminogroups connected to the conjugated molecular system by analogy with aromatic amines, along with sulfo- and seleno-groups, may serve as additional antiradical centers [15].

The present study was aimed at investigating the antiradical activity and capacity of seleno-organic and sulfo-organic compounds: selenophene, thiophene and their aminocarbonitrile derivatives. Among the chosen methods, oxygen radical absorbance capacity (ORAC) is a key one which makes it possible to investigate the antiperoxyradical capacity of compounds under consideration. The obtained information allows to estimate their antioxidant capacity acting as a chain break mechanism in the reactions of lipid peroxidation of cell membranes.

Selection of the 2,2ʹ-diphenyl-1-picrylhydrazyl (DPPH) kinetic method combined with differential pulse voltammetry (DPV) pursues the following objectives:

- To reveal the difference of antiradical capacity by means of assessing radicals with different nature: DPPH [16] and peroxyradicals.
- Using the DPV voltammetry method to characterize quantitively the redox properties of the studied sulfur- and selenium-containing compounds, as well as to find a correlation with their antiradical properties.

## 2. Materials and Methods

### 2.1. Materials

Selenophene and thiophene derivatives, ATS and ATSe, were synthesized by the method described in [17,18]. Azoinitiator 2,2ʹ-azobis(2-amidinopropane) hydrochloride (AAPH), 6-hydroxy-2,5,7,8-tetramethylchromane-2-carboxylic acid (Trolox), butylatedhydroxytoluene (BHT), 3ʹ,6ʹ-dihydroxyspiro[isobenzofuran-1(3H), 9ʹ-[9H]xanthen]-3-one-fluorescein disodium salt (Fl), DPPH, selenophene, and thiophene were purchased from the Sigma-Aldrich (USA) chemical company. Other chemicals—tetrabutylammonium perchlorate, and silver nitrate (AgNO_3_)—and solvents—acetonitrile (ACN), ethanol (C_2_H_5_OH), methanol (CH_3_OH), and phosphate buffer—were also purchased from the same company and were purified by the method described in [19] (except phosphate buffer). In all experiments, deionized water with electrical resistance 16 MOm·sm at 25 °С was used.

### 2.2. Oxygen Radical Absorption Capacity (ORAC) Method

The ORAC method is based on measuring the fluorescein fluorescence intensity decrease vs. time as a result of the reaction with peroxyradicals [20,21]. One of the features of the method is the possibility to determine antiperoxyradical capacity in aqueous medium. For this purpose, a Perkin-Elmer fluorescence spectrometer MPF-44B (USA) was used, with the computer recording the kinetics of the Fl fluorescence intensity. The wavelengths of excitation and emission light were 450 and 515 nm, respectively. Quantitative determination of the antiperoxyradical capacity of the studied compounds was carried out by calculating the area between the two decay curves (with or without antioxidant) in conditions of full consumption of Fl. A water-soluble analogue of α-tocopherol, Trolox, served as the standard antioxidant.

The value of the antiperoxyradical capacity, *f*_AO_, was determined by the following equation [20]:

(1) $$f_{\mathrm{AO}}=\frac{\left(S_{\mathrm{AO}}{-S}_{0}\right)M_{\mathrm{trolox}}}{\left(S_{\mathrm{trolox}}{-S}_{0}\right)M_{\mathrm{AO}}}{\text{×}f}_{\mathrm{trolox}}$$

where *S*_0_, *S*_AO_ and *S*_trolox_ are the areas between the kinetic curves of fluorescence intensity decrease with and without the studied antioxidants and with the standard antioxidant, Trolox, respectively; *M*_trolox_, *M*_AO_ are the molar concentrations of Trolox and the antioxidants under study, respectively; *f*_AO_, *f*_trolox_ are the antiperoxyradical capacities of the antioxidants and Trolox, indicating the number of radicals scavenged by an antioxidant molecule.

**Scheme 2:**
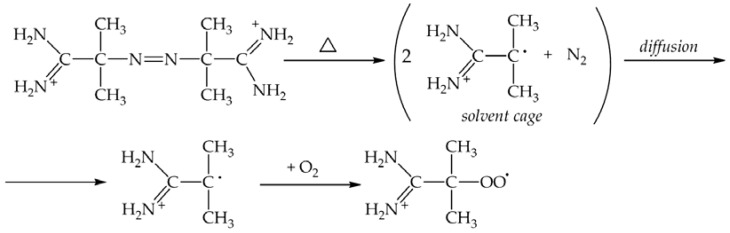
Generation of peroxyradicals by thermal decomposition of the water-soluble azo-compound AAPH.

Antiperoxyradical capacity relative to Trolox equivalent (*f*_REL,TE_) is calculated as *f*_REL,TE_ = *f*_AO_/*f*_trolox_. Integration of appropriate areas is performed by the trapezoid method using Microcal Origin 8.0 graphing and data analysis software (OriginLab Corporation, Northampton, Massachusetts, USA) [22]. Peroxyradicals were generated by the thermal decomposition of the water-soluble azoinitiator, AAPH, at temperature 37 °С (Scheme 2).

### 2.3. Electrochemical Measurments

The following versions of the voltammetry method: differential pulse (DPV), cyclic (CV) and squarewave (SWV), were used. They were applied by means of the Bioanalytical system BAS-100B/W (USA) using three-electrode configuration. The working electrode is made of glassy carbon (surface ~ 0.09 cm^2^). Before each measurement, the electrode was purified by Al_2_O_3_ powder with a particle size of 0.5 microns for 3 min. The Ag/AgCl/KCl system served as a reference electrode, and a platinum electrode served as an auxiliary one. During CV, DPV and SWV measurments of the studied antioxidants, tetrabutylammonium perchlorate in ACN with the concentration of 0.1 M was used as a background electrolyte. Concentrated solutions of 4 × 10^−4^ M of selenophene and aminocarbonitrile derivatives, ATS and ATSe, were prepared in ethanol, and a concentrated solution of thiophene (4 × 10^−4^ M) was prepared in methanol.

In the experiments, solutions were saturated by molecular nitrogen (99.99%) for 10 min before measuring. Test experiments with the electrochemical analysis system were carried out using standard solutions of Na_3_Fe(CN)_6_. At calibration, the linear correlation coefficient was 0.9995. The volume of the electrochemical cell was 10 ml, the temperature was 37 ± 0.1 °C, and the rate of voltage scans was 20 mV/s. In the DPV case, the pulse amplitude was 50 mV, the pulse duration was 50 ms, and the pulse period was 200 ms. In the case of SWV, the frequency was 25 Hz, and the squarewave amplitude was 25 mV, for the CV case, the sweep segment was 2. CV, DPV, SWV voltammograms were recorded from −1200 to +1600 mV.

Reactions of sulfur- and selenium-containing compounds with DPPH radicals were studied by the DPV method using DPPH as an analytical reagent. Within the studied interval of DPPH concentrations, a linear relation between the respective anodic oxidation current and the concentration of DPPH (with a correlation coefficient 0.9912) was observed.

The antiradical capacity of antioxidants using DPPH were calculated by the following formula:

(2) $$f_{\mathrm{DPPH}}=\frac{{\left[\mathrm{DPPH}\right]}_{0}-{\left[\mathrm{DPPH}\right]}_{\infty }}{{\left[\mathrm{AO}\right]}_{0}}$$

where [DPPH]_0_ and [AO]_0_ are the initial concentrations of DPPH and the studied antioxidant, respectively; [DPPH]_∞_ is the residual concentration of DPPH after complete consumption of the antioxidant as a result of the reaction. Calculation of *f*_DPPH_ was performed on the basis of kinetic curves of DPPH reactions with antioxidants.

## 3. Results

**Figure 1:**
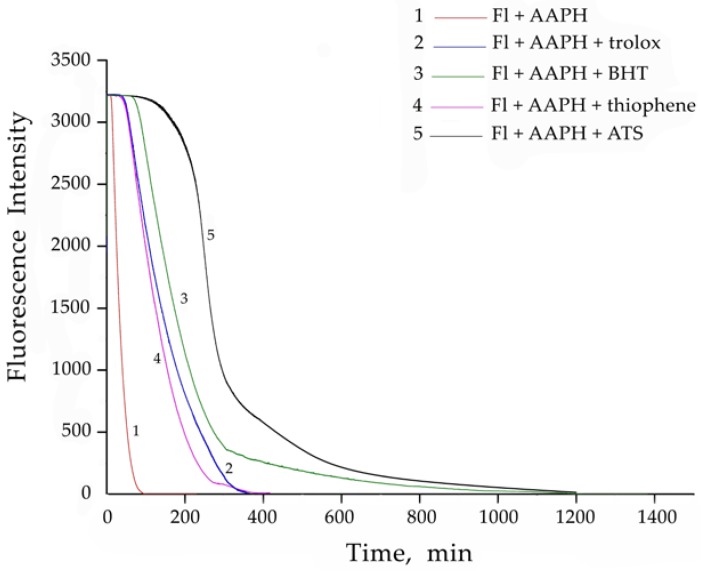
Kinetic curves of Fl fluorescence intensity at 515 nm decrease vs. time as a result of the reaction with peroxyradicals in the absence (1) and presence of antioxidants: Trolox (2), butylatedhydroxytoluene (BHT) (3), thiophene (4) and ATS (5) at 37 °С. [AAPH]_0_ = 1.53 × 10^–2^ M (radical initiation rate was equal to *R*_i_ = 3.2 × 10^−8^ M·s^−1^), antioxidants’ concentration was 5 × 10^−6^ M. Deionized water served as a solvent.

**Figure 2:**
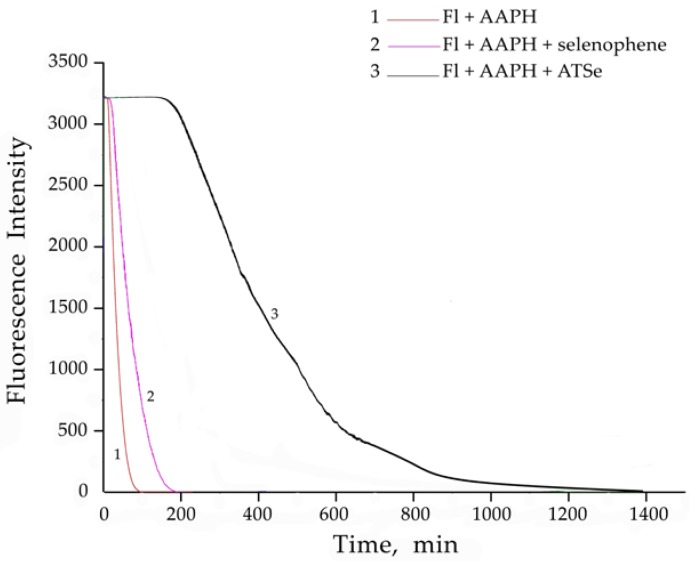
Kinetic curves of Fl fluorescence intensity at 515 nm decrease vs. time as a result of the reaction with peroxyradicals in the absence (1) and presence of antioxidants: selenophene (2) and ATSe (3) at 37 °С. [AAPH] = 1.53 × 10^−2^ M (radical initiation rate was equal to *R*_i_ = 3.2 × 10^−8^ M·s^−1^), antioxidants’ concentration was 5 × 10^−6^ M. Deionized water served as a solvent.

On the basis of ORAC kinetic measurements (Figure 1 and Figure 2) and using Equation (1), the antiperoxyradical capacities of the studied antioxidants were calculated and the results are summarized in Table 1.

**Figure 3:**
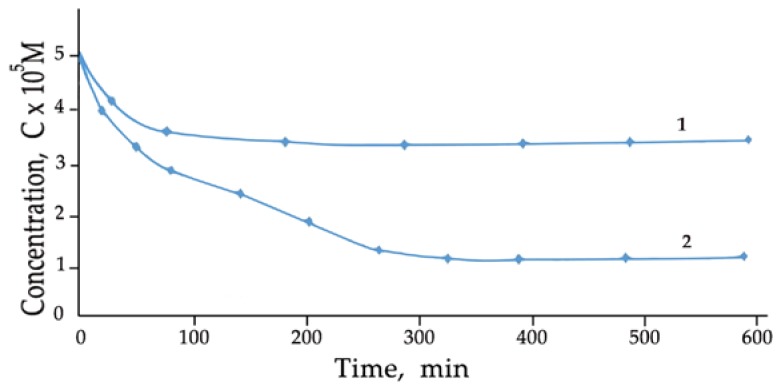
Kinetic curves of 2,2ʹ-diphenyl-1-picrylhydrazyl (DPPH) concentration decrease as a result of the reaction with thiophene (1) and ATS (2). [thiophene]_0_ = 2.5 × 10^−5^ M, [ATS]_0_ = 2.5 × 10^−5^ M, [DPPH]_0_ = 5 × 10^−5^ M at 37 ^°^С. The differential pulse voltammetry (DPV) method was used.

**Figure 4:**
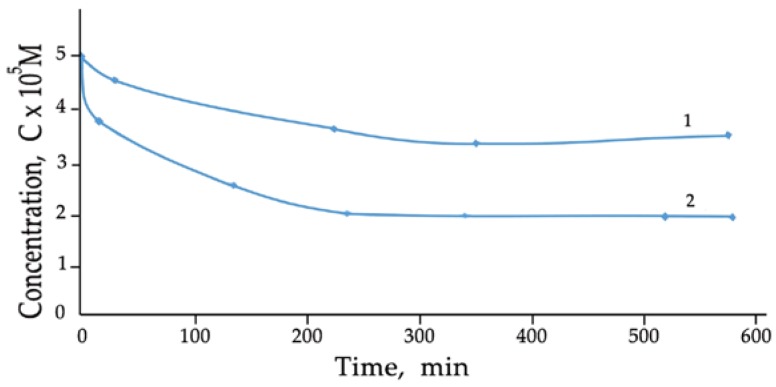
Kinetic curves of DPPH concentration decrease as a result of the reaction with selenophene (1) and ATSe (2). [selenophene]_0_ = 2.5 × 10^−5^ M, [ATSe]_0_ = 2.5 × 10^−5^ M, [DPPH]_0_ = 5 × 10^−5^ M at 37 °С. The DPV method was used.

The antiradical capacities of the same antioxidants in relation to DPPH were calculated according to the kinetic curves demonstrated in Figure 3 and Figure 4 and using Equation (2).

As can be seen from the data obtained, the antiradical capacities of thiophene and selenophene are smaller compared to other antioxidants, including Trolox and BHT. The *f*_REL,TE_ value for thiophene is greater than that of selenophene.

**Figure 5:**
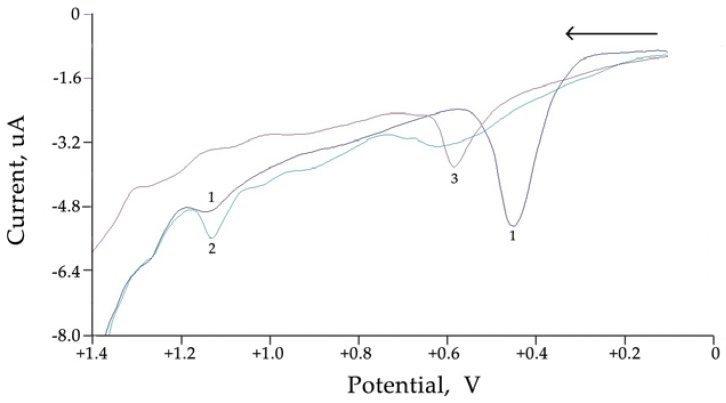
DPV voltammograms for DPPH (1) and antioxidants: thiophene (2), ATS (3) at 37 °С. [DPPH]_0_ = 5 × 10^−5^ M, [thiophene]_0_ = 2.5 × 10^−5^ M, [ATS]_0_ = 2.5 × 10^−5^ M.

**Figure 6:**
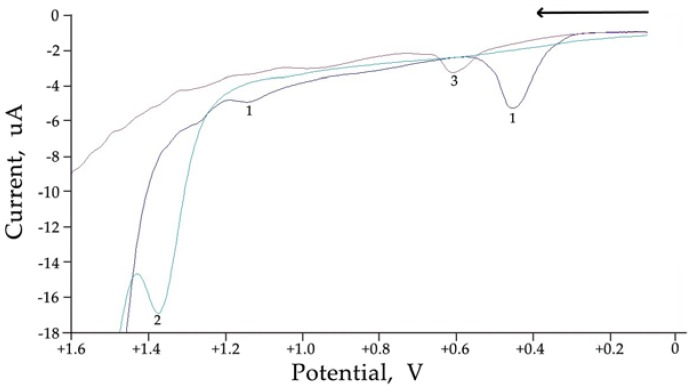
DPV voltammograms for DPPH (1) and antioxidants: selenophene (2), ATSe (3) at 37 °С. [DPPH]_0_ = 5 × 10^−5^ M, [selenophene]_0_ = 2.5 × 10^−5^ M, [ATSe]_0_ = 2.5 × 10^−5^ M.

The typical DPV voltammograms which were used to determine the kinetics of the DPPH reaction with antioxidants are given in Figure 5 and Figure 6. At the same time, in these figures, anodic oxidation peaks of the studied seleno- and sulfo-organic compounds are illustrated.

In general, values of the characteristic peaks of oxidation and reduction of the studied antioxidants by various voltammetric methods are given in Table 2.

The relatively lower antiradical capacity of selenophene and thiophene compared with their aminocarbonitrile derivatives correlates with their lower characteristic values of anodic oxidation peaks (Table 2, Figure 3 and Figure 4). At the same time, lower initial rates of DPPH consumption in the reaction with thiophene and selenophene were observed (in Figure 5 and Figure 6). In this case, the initial rates of DPPH consumption characterize the antioxidant activity of the studied compounds.

Meanwhile, for the organic aminocarbonitrile derivatives of both selenophene and thiophene, very high values of antiperoxyradical capacity, 7.78 and 5.82, respectively, were observed. Moreover, the highest value of antiradical capacity was registered for the selenium derivative ATSe. Usually, the aminogroup in the conjugated ring exhibits high antiradical activity [23]. These data correlate with antiradical capacity and activity data, determined by using DPPH. The difference between the absolute values of *f*_AO_ and *f*_DPPH_ is due to the nature of radicals—active peroxyl radical (ROO^•^) and significantly less active DPPH.

It is supposed that sulfur and selenium atoms in the molecular structure of selenophene and thiophene are responsible for their antiradical ability, by analogy with dimethylselenoxide [24]. In this case, sulfur and selenium atoms in the molecules of selenophene and thiophene can act as four-electron reducing agents. It is well-known that the peroxyl radical (ROO^•^) is most often a one-electron oxidant. So, it can be expected that one molecule of these antioxidants is capable of capturing four peroxyl radicals. Meanwhile, their smaller values of *f*_AO_ that were observed are presented in Table 1. This may be due to the fact that, in parallel with the capture of peroxyl radicals by these compounds, the two-electron oxidation reaction of antioxidants byperoxyl radicals can occur, resulting in the generation of a new active alkoxyl radical RO^•^. Further, RO^•^ may directly interact with both Fl or seleno- and sulfo-organic compounds. Thus, one molecule of ROO^•^ may lead to two- or three-electron oxidation of selenium- and sulfur-containing compounds. As a result, the antioxidant capacity of Se and S atoms against molecules decreases, as is shown in Table 1.

**Scheme 3:**
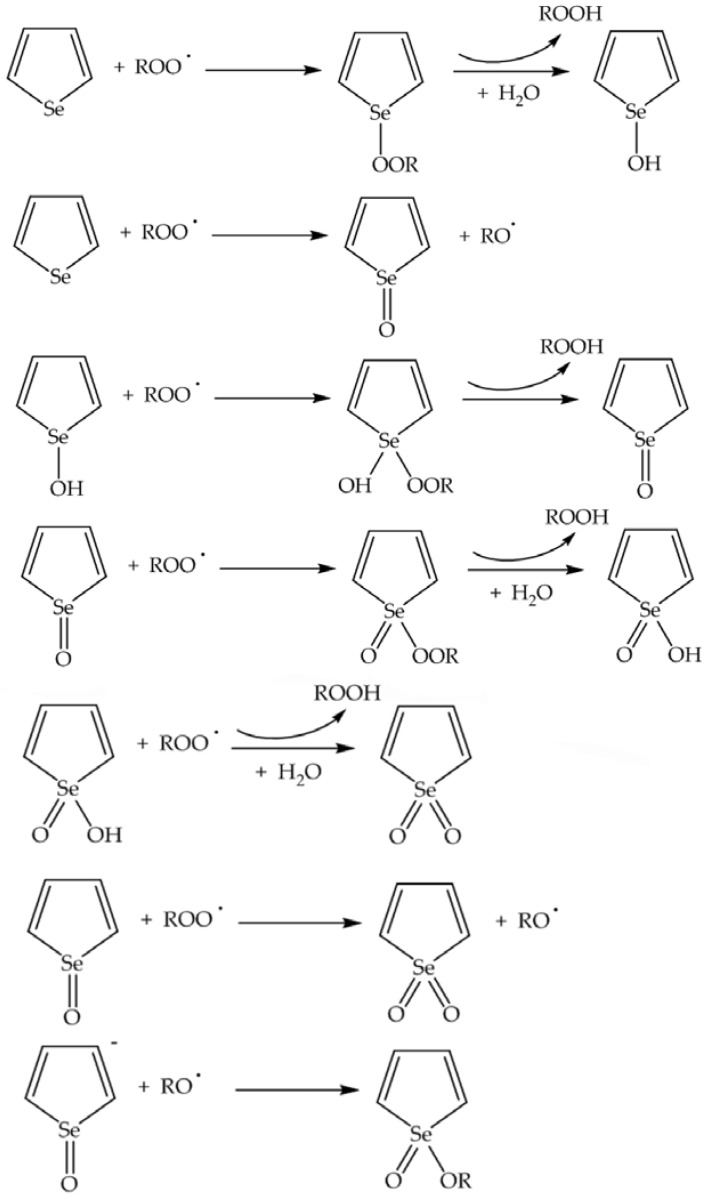
Proposed chemical transformation sequence of selenophene in reactions with peroxyl radicals.

The proposed mechanism of antiperoxyradical ability by the example of selenophene is presented in Scheme 3.

**Scheme 4:**
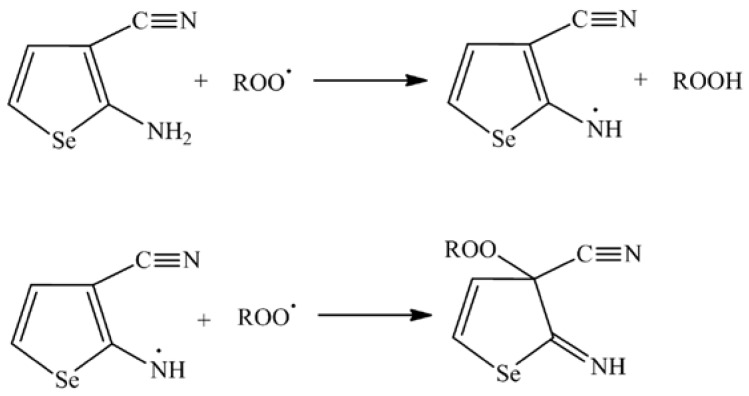
Capture of peroxyl radicals by ATSe as a result of the reaction with aminogroup.

By analogy with aromatic amines [15,23], the aminogroup of thiophene and selenophene aminocarbonitriles may exhibit antiperoxyradical activity as a result of reactions shown in Scheme 4:

Thus, there are two reasons leading to the significant increase in the antiradical capacity in the case of the aminocarbonitrile derivatives of thiophene and selenophene.

(1) Additional scavenging of free radicals by the aminogroup. For aromatic amines with a monoamine group, the value of *f*_AO_ is 2–3 [23].
(2) The amino group may significantly reduce the portion of reactions involving cyclic chalcogen atoms and peroxyl radicals forming an active alkoxy radical, which result in decreasing the effective value of antioxidant capacity (*f*_AO_).

## 4. Conclusions

In conclusion, it should be mentioned that thiophene, selenophene and their aminocarbonitrile derivatives exhibit antiradical ability. Moreover, the antiradical capacity of thiophene, selenophene and their aminocarbonitrile derivatives significantly exceeds that of their simple analogues, and the traditional antioxidants, Trolox and BHT.

The data obtained allow one to expect that thiophene and selenophene aminocarbonitrile derivatives, as well as newly synthesized organic compounds on their base, may be potential agents against the pathologies of oxidative stress.

## Figures and Tables

**Table 1:** Antiradical capacities of antioxidants.

Antioxidant	*f*_(REL,TE)_	*f*_AO_	*f*_DPPH_
thiophene	0.85	1.71	0.46
ATS	2.91	5.82	1.46
selenophene	0.30	0.61	0.52
ATSe	3.89	7.78	1.26
BHT	1.29	2.58	-
trolox	1	2	0.79

It was supposed that *f*_trolox_ = 2—on the basis of the fact that Trolox scavenges 2 peroxyl radicals—one in the initial form, and the second formed by tocopheroxyl radical.

**Table 2:** Values of the characteristic peaks of oxidation and reduction of thiophene, selenophene, ATS, ATSe, BHT and Trolox, obtained by voltammetric methods: DPV, cyclic voltammetry (CV) and squarewave voltammetry (SWV). The sign “-“ indicates that measured peaks were not observed.

Voltammetric Method		ATS	ATSe	Thiophene	Seleno-phene	Trolox	BHT
DPV	oxidation	584 mV	608 mV	1128mV	1372 mV	244 mV	508 mV
	reduction	620 mV	621 mV	1245mV	1056 mV	1016 mV	1088 mV
CV	oxidation	621 mV	620 mV	-	-	-	-
	reduction	675 mV	615 mV	-	-	-	106 mV
SWV	oxidation	612 mV	616 mV	-	1420 mV	-	-
	reduction	636 mV	632 mV	-	-	-	-
